# Solved the enigma of pediatric severe acute hepatitis of unknown origin?

**DOI:** 10.3389/fcimb.2023.1175996

**Published:** 2023-09-21

**Authors:** Francisco Rodriguez-Frias, Ariadna Rando-Segura, Josep Quer

**Affiliations:** ^1^Clinical Biochemistry Department Vall d’Hebron Institut of Research (VHIR), Vall d’Hebron Barcelona Hospital Campus, Barcelona, Spain; ^2^Basic Science Department, International University of Catalonia, Barcelona, Spain; ^3^Centro de Investigación Biomédica en Red de Enfermedades Hepáticas y Digestivas (CIBERehd), Instituto de Salud Carlos III, Madrid, Spain; ^4^Liver Diseases-Viral Hepatitis, Liver Unit, Vall d’Hebron Institut de Recerca (VHIR), Vall d’Hebron Barcelona Hospital Campus, Barcelona, Spain; ^5^Biochemistry and Molecular Biology Department, Autonomous University of Barcelona (UAB), Barcelona, Spain

**Keywords:** acute hepatitis, severity, SARS-CoV-2, adenovirus, adeno-associated virus, liver transplant, mortality

## Abstract

Hepatitis is an inflammation of the liver whose etiology is very heterogeneous. The most common cause of hepatitis is viral infections from hepatotropic viruses, including hepatitis A, B, C, D and E. However, other factors such as infections from other agents, metabolic disorders, or autoimmune reactions can also contribute to hepatitis, albeit to a lesser extent. On April 5, 2022, the United Kingdom Health Security Agency alerted the World Health Organization (WHO) on the increased incidence of severe acute hepatitis of unknown causes (not A-E) in previously healthy young children, with symptoms of liver failure that in some cases required liver transplantation. By July 2022, 1,296 cases were reported in 37 countries. Acute hepatitis of unknown causes is not an exceptional phenomenon: in fact, it represents more than 30% of cases of acute hepatitis in children, however in the present instance the large proportion of severe cases was surprising and alarming (6% of liver transplants and almost 3% mortality). Multiple hypotheses have been proposed to explain the etiology of such higher proportion of acute hepatitis, including their co-occurrence in the context of COVID-19 pandemic. This is a review of the history of a clinical threat that has put in check a world health care system highly sensitized by the current COVID-19 pandemics, and that it looks like has ended with the arguments that the severe acute pediatric hepatitis is caused by Adeno-associated virus 2 (AAV2) infection associated with a coinfection with a helper virus (human Adenovirus HAdV or human herpesvirus 6) in susceptible children carrying HLA-class II antigen HLA-DRB1*04:01.

## Introduction

Hepatitis is an inflammation of the liver, which can affect the portal tract or the hepatic acinus or combine both phenotypes. The causes of hepatitis are multi etiological. Initially, a possible infection by hepatotropic viruses, such as hepatitis viruses A, B, C, D and E (not A-E) was proposed as a main cause, however other infectious agents may also be responsible in a minority of patients (**Box 1**). Liver damage can also arise as a post-toxin event to drugs or botanicals. Other events may include excessive accumulation of fat in the hepatocytes, or NAFLD (non-alcoholic fatty liver disease)/NASH (non-alcoholic fatty liver disease-steato-hepatitis), or autoimmunity/immune dysregulation. The diagnosis of acute hepatitis of unknown origin not A-E generally refers to liver injury caused by the exclusion of known infectious or non-infectious factors (**Box 1**). This disease represents over 30% of the cases of acute hepatitis in children (Alonso et al., 2017).

Box 1 **Definition of cases to report and guidelines for ruling out known etiologies.**

**Definitions of cases.** ECDC case definition. (a) *Confirmed*: A person presenting with an acute hepatitis (non hepA-E*) with serum transaminase >500 IU/l (Aspartate Transaminase-AST or Alanine Transaminase-ALT), who is 10 years and under, since 1 January 2022. (b) *Possible*: A person presenting with an acute hepatitis (non hepA-E*) with serum transaminase >500 IU/l (AST or ALT), who is 11 to 16 years, since 1 January 2022. (c) *Epi-linked:* A person presenting with an acute hepatitis (non hep A-E*) of any age who is a close contact of a confirmed case, since 1 January 2022. (*) Cases with other explanations must be discarded. WHO case definition. (a) *Confirmed case*: not available. (b) *Probable case*: A person with acute hepatitis (not A, B, C, D, E*) with serum transaminase levels >500 IU/L (AST or ALT). 16 years of age or younger, as of October 1, 2021. (*) Cases with other explanations must be discarded.

**Guidelines for ruling out know etiologies**. (1) PCR tests using blood/serum samples for adenovirus, enterovirus, human herpes virus 1, 2, 3, 4, 5, 6 and 7, hepatitis A, C and E virus; (2) Serological tests for hepatitis A, B, C, and E viruses, EBV, and cytomegalovirus (CMV) in addition to SARS-CoV-2; (3) Blood culture for bacteria if fever is present; (4) Panel of multiplex PCR respiratory viruses (including adenovirus, enterovirus, influenza virus, human bocavirus, and SARS-CoV-2) from the earliest possible throat swab; (5) Multiplex PCR gastrointestinal virus panel (including adenovirus, sapovirus, norovirus, enterovirus) in a stool sample; (6) Culture of stool for common bacterial enteropathogens, including Salmonella; (7) Test for adeno-associated and Adenovirus co-infection.

Serologic testing for anti-streptolysin O (ASO), throat swab culture for group A hemolytic streptococci, and serum/urine tests for leptospirosis should be considered if clinically indicated. Toxicology screening with blood and urine samples should also be considered.

## A new health alarm in children: another *deja vu* feeling or it is actually happening?

Today, scientific and technological advancements have reached unparalleled levels of precision, sophistication, and power, enabling us to investigate diseases that occurred in ancient times, long before historical records were even conceived (Rodríguez-Frías et al., 2021). These extraordinary advances in technology and communication, coupled with the researchers’ ability of channeling their creativity, have fostered true neural networks among professionals worldwide. Irrespective of any alarming situation, this interconnected global medical community can be used to face present and future unknown clinical scenarios.

One such scenario emerged on April 5, 2022, when the United Kingdom Health Security Agency (UKHSA) alerted the World Health Organization (WHO) about a notable increased incidence of severe acute hepatitis of unknown cause among young children (UKHSA, 2022). Almost simultaneously, a similar situation was reported at the Children’s Hospital of Alabama (USA), where the majority of children admitted with acute hepatitis had an unknown cause (Gutierrez Sanchez et al., 2022), raising significant concerns due to the severity of its clinical presentation and the young age of the affected children. The number of admissions in 2022 in UK, was equal or greater than the total annual admissions in previous years. Similarly, the Women’s and Children’s Hospital in Alabama (USA) reported an increase, with 10 cases admitted between January and March 2022 compared to 1 to 5 cases per year previously (Kelgeri et al., 2022).

Consequently to these observations, the European Center for Diseases Prevention and Control (ECDC) and the Center for Disease Control and Prevention (CDC) issued a warning about hepatitis of unknown origin in children (who/ecdc, 2022; World Health Organization (WHO), 2022). Of the 13 cases notified by the British agency to the WHO, 10 cases aroused in Scotland in children of 11 months to 5 years of age and required hospitalization. One child had an onset of symptoms in January 2022 and nine cases on March 2022. The children presented frequent non-specific gastrointestinal symptoms, such as diarrhea and vomiting, progressing to jaundice, abdominal pain, nausea, and malaise, with elevated aminotransferase (ALT) and bilirubin levels. On April 8, 2022, an UK nationwide investigation identified a total of 74 cases with similar characteristics to the original 10 cases, including high aminotransferase levels (>500 IU/L) in serum, being negative for hepatitis A to E virus infections. Conceivably, patients with milder hepatitis may have not been detected and therefore notified.

In light of the aforementioned scenarios, WHO, ECDC, CDC, and UKHSA agencies reached a consensus on the definition for the acute hepatitis, similar to the one employed in Scotland, based on exclusion of other causes of acute hepatitis. In this new definition, the category of “confirmed case” was replaced by “probable case” (**Box 1**). It should be noticed that all documented cases coexisted with previously reported cases, including those diagnosed under different criteria. Between April and September 14, 2022, a total of 1,296 probable cases had been reported from 37 countries/regions (**Table 1**), 40% of them (519) were from Europe, predominantly from Scotland and the UK (277), while 28% were from US (364), with approximately 55 cases requiring liver transplantation and 29 resulted in death (Gong et al., 2022). These data, along with the ongoing prevalence of SARS-CoV-2 infection, led the cases of severe acute hepatitis in children be considered a potentially new “health alarm”.

**Table 1:** Distribution of the 1296 cases reported up to July 12, 2022.

Region	Number of cases	Required Liver Transplant	SARS-CoV-2 positive by PCR	Adenovirus positive by PCR	Adenovirus type 41	Deaths
**America**	635	26	18/222	144/325	14/28	14
**Europa**	519	28	56/376	225/410	62/70	3
**Asia**	142	1	6/62	7/62	0/3	12
**TOTAL**	**1296**	**55**	**78**	**209**	**31**	**22**

https://www.who.int/emergencies/disease-outbreak-news/item/2022-DON400#:~:text=Severe%20acute%20hepatitis%20of%20unknown%20aetiology%20in%20children%20%2D%20Multi%2Dcountry,-12%20July%202022&text=As%20of%208%20July%202022,case%20definition%2C%20including%2022%20deaths.

In the last decade the evident success of vaccinations against hepatitis A and B viruses has caused a great reduction of acute infections of known origin. Nonetheless, certain viruses other than the classical hepatotropic ones (A to E) that are frequent in childhood, also present a certain hepatotropism, such as Epstein-Barr Virus (EBV), herpes simplex virus; varicella-zoster virus; human herpesvirus 6, 7, and 8; human parvovirus B19; adenoviruses; cytomegalovirus; among others (Gallegos-Orozco and Rakela-Brödner, 2010). These viruses together with other possible non-viral, toxic or autoimmune dysfunctions may cause a severe disease in a minority of patients (**Box 1**). In such cases, liver damage is very rapid and it is accompanied by important elevations of liver enzymes (over 100 times the upper limit of the normal range) ALT and aspartate aminotransferase (AST). In these severe cases, patients experience a rapid deterioration in their condition, resulting in significant liver function impairments such as coagulopathy, jaundice, and encephalopathy, which can ultimately progress to liver failure. In rare instances, liver failure may require transplantation, and in many patients with functional deficiencies no marked elevations of aminotransferases are observed and most of them recover due to the remarkable regenerative capacity of the liver.

In large series of children hospitalized with liver failure, most of them (between 49-64.5%) met the criteria of pediatric acute liver failure of unknown etiology or indeterminate (Squires et al., 2006; Squires et al., 2014; Leiskau et al., 2023). In a 2017 study from the Pediatric Acute Liver Failure Study Group (PALFSG) including over 1,000 children, 30% of miss-diagnosis were reported with more than 60% of cases observed in children of 1 to 5 years of age (Alonso et al., 2017). Given the extraordinary youth of the affected individuals, any possible cause should be explored including genetic and environmental triggers, able to explain the most severe cases(Centers for Disease Control and Prevention (CDC), 2022; Squires et al., 2022). As previously stated, although rare, acute hepatitis of unknown etiology in children is not a new entity, but the current alarming situation seems truly exceptional.

**Figure 1:**
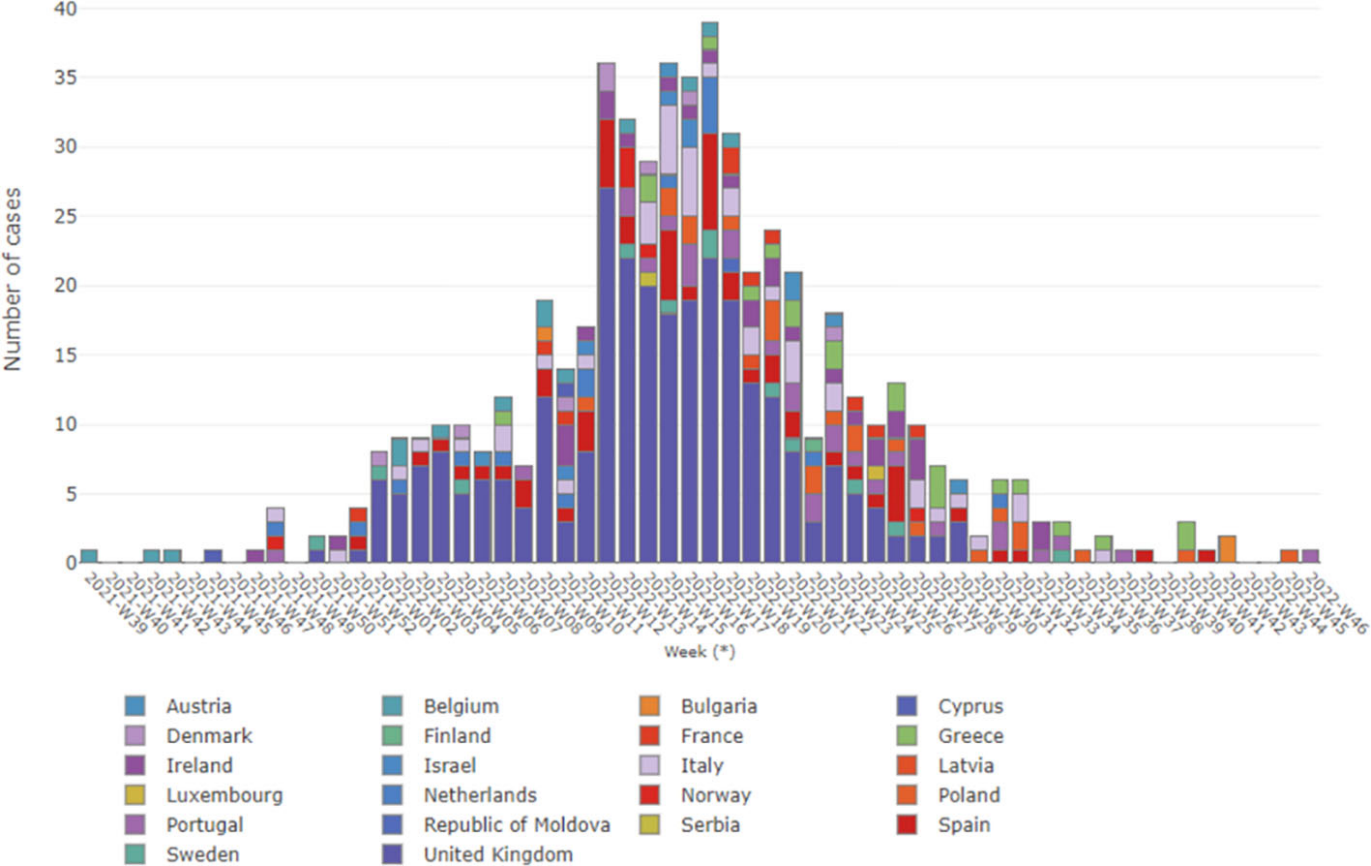
Severe acute hepatitis in children, in Europe WHO region, by week. Data updated up to 27 October 2022, and obtained from WHO (World Health Organization (WHO), 2022).

Currently, while the situation of acute hepatitis of unknown etiology in children remain as “open active alert”, we consider helpful to provide an update as of October 27, 2022, specially focusing on the WHO European Region having important repository Historical Archives. Considering the 22 countries (HAEC 2022), 563 cases have been reported: Austria (6), Belgium (14), Bulgaria (1), Cyprus (2), Denmark (8), Finland (1), France (10), Greece (20), Ireland (29), Israel (5), Italy (47), Latvia (1), Luxembourg (1), Netherlands (16), Norway (6), Poland (22), Portugal (26), Republic of Moldova (1), Serbia (1), Spain (54), Sweden (12) and the United Kingdom (280) (Ministerio de Sanidad, 2022; who/ecdc, 2022). The epidemiological curve shows the cases by date of symptoms onset (381 cases), date of hospitalization (159 cases) and date of notification at the national level. The cases (numbers) by week in Europe as of October 27 are shown in **Figure 1** (Pan et al., 2022; World Health Organization (WHO), 2022), showing that there was a notable increase in cases from week 12, remaining stable until week 18 with 28 to 39 cases reported per week. Although late reporting may affect recent case numbers, there was a steady decline in weekly reported cases starting from week 18. The majority of patients (75.7%) were five years old or younger. In Europe, there were seven deaths related to the disease out of the 364 reported cases. Among the reported cases, 98 (26.9%) required admission to the Intensive Care Unit. Out of the 313 cases with available information, 24 cases (7.7%) underwent a liver transplant. Among 440 cases analyzed for different adenovirus detection, 231 (52.5%) tested positive. The highest positivity rate was observed in whole blood samples (49.5%). Adenovirus typing information was only available for eleven cases: type 31 (n = 1), type 40 (n = 3), type 41 (n = 5) and other types (n = 2). Among 384 cases analyzed for SARS-CoV-2 detection using PCR, 40 (10.4%) were positive. However, serology results for SARS-CoV-2 were available for only 109 cases, with a higher frequency of 68 (62.4%) positive cases. In addition, out of 162 cases with vaccination data against COVID-19, 143 (88.3%) were not vaccinated (Ministerio de Sanidad, 2022).

A global predisposition based on gender and ethnicity was observed. In Spain, the Health Alerts and Emergencies Coordination Center (HAECC) issued a report on April 22, 2022, regarding “Alert of severe acute hepatitis of unknown causes, non A-E, in children under 10 years in UK. Situation in Spain”. The report stated that as of November 10, there were 59 unrelated cases of liver failure in children under investigation in Spain, with 48 of them being 10 years old or younger. No cases with epidemiological link were found. A microbiological study of 59 cases, confirmed the absence of known causes of hepatitis in 32 of them (viruses A to E, leptospira, parvovirus B19, herpes simplex, and varicella zoster virus, were negative) (Ministerio de Sanidad, 2022). When the 32 cases were analysed for herpes virus, 8 patients (25%) were found positive for herpes viruses, 6 of them for cytomegalovirus (CMV), 1 was herpes type 6 and the last was epstein-barr virus (EBV) plus herpes type 7. Additionally, 15 out of the 32 cases (47%) were positive for adenovirus, and 3 cases resulted positive for enterovirus in serum (one of them typed by metagenomics as coxsackie B4). Further analysis of the 15 adenovirus-positive cases revealed infection of type-2 adenovirus (1 case), and type-41 adenovirus (1 case) using metagenomics. In addition, 4 other adenoviruses (two types 5 and two types 41) were identified using PCR/partial sequencing. In addition, using metagenomics, 8 cases of adenovirus revealed the adeno associated, dependovirus-parvovirus A (AAV), and 2 cases were AAV type 2. Interestingly, AAVtype 2 has not been previously associated with any human pathology. Additionally, other viruses of known pathogenic interest have been found: 1 coronavirus NL63, 1 echovirus 11, 3 sapporo virus, and 2 parechovirus. Multiple pathogens were found in 15 cases, while no pathogens were found in 10 cases. The HAECC report in Spain concluded that adenovirus was commonly detected in severe acute hepatitis of unknown causes.

It is worth mentioning that the liver histology of autoimmune hepatitis is very similar to that induced by drugs or toxins, characterized by the presence of eosinophils. Hepatitis has been reported as part of a multisystem inflammatory syndrome in children (MIS-C) (Heinz et al., 2020; Xu et al., 2020). While it is well-known that influenza viruses, coronavirus, herpesvirus, CMV and EBV virus can cause hepatitis in both immunocompetent and immunocompromised adults and children (Spengler, 2020), none of the aforementioned viruses can be associated with the current case of severe acute hepatitis in pediatric patients. Regarding the management of clinical patients, the majority of children with severe acute hepatitis typically achieve full recovery with supportive care. However, it is important to acknowledge that the clinical course of the disease can be unpredictable, and in rare instances, it may progress to acute liver failure. Therefore, it is crucial for clinicians to be well-informed about the signs that indicate disease severity progression and to stablish a threshold for referring patients to a liver transplant center (Leiskau et al., 2023) (**Box 1**).

## Was SARS-CoV-2 infection a critical cofactor for the development of severe acute hepatitis in children?

The prevalence of induced hepatic dysfunction including “cytokine storm” injury (Li and Fan, 2020; Trevenzoli et al., 2020), increased susceptibility to HAdVs contagion or a greater virulence of the adenovirus caused by the concurrent SARS-CoV-2 infection(Brodin and Arditi, 2022), hyper immunization-related factors (Avci and Abasiyanik, 2021; Bril et al., 2021; Rocco et al., 2021; Vuille-Lessard et al., 2021), molecular mimicry (Lai et al., 2022), and auto-inflammatory dysregulation caused by the virus itself (Vojdani and Kharrazian, 2020; Bril et al., 2021; Rocco et al., 2021; Vuille-Lessard et al., 2021), have supported a potential relationship between SARS-CoV-2 infection and the severe acute hepatitis in children. However, only 15% of the children with available data in Europe and 10% of cases in USA, meet the conditions (World Health Organization (WHO), 2022). For example, in the previously mentioned series of 44 children from the same hospital in Birmingham, Al, USA, only one child tested positive for SARS-CoV-2, 6-8 weeks prior a seizure episode (Kelgeri et al., 2022). For acute hepatitis, only 11 of 39 children were positive for SARS-CoV-2 at admission.

The increase in severe acute hepatitis coincided with the moment in which the Delta variant alternated with the Omicron variant. The latter was described to cause gastrointestinal symptoms in children, such as vomiting, diarrhea, abdominal pain, and anorexia, suggesting an increase in intestinal tropism of SARS-CoV-2 that overlaps with the described ACE2 expression in the small intestine (Zhang et al., 2022). Interestingly, despite the high expression of ACE2 in hepatocytes and cholangiocytes, and that the viral tropism for the liver and the underlying mechanisms have been stablished (Wanner et al., 2022), the hepatic involvement of COVID-19 is very rare (Louis et al., 2022). This observation appears to rule out a direct implication of Omicron infection and non A-E acute hepatitis cases. However, concomitant infections of Omicron with HAdV-F41, or Adeno Associated Virus 2 (AAV2) was proposed by Grand (Grand, 2022). An attractive hypothesis suggests that SARS-CoV-2 may be acting as a superantigen, suggesting that these cases of severe acute hepatitis in children infected by SARS-CoV-2 could be caused by a previous HAdV-F41 infection in the intestine (Brodin and Arditi, 2022). This idea stems from the description of cases where severe hepatitis resulted from a multisystem inflammatory syndrome (MIS) observed in children with COVID-19 (Cantor et al., 2020). However, MIS typically occurs 3-4 weeks after the peak of COVID-19, and 60% of seropositive children with MIS have no detectable virus, indicating that MIS may result from the immune response following the infection (Verdoni et al., 2020). It is worth noting the persistence of SARS-CoV-2 viral RNA in the gastrointestinal tract of children compared to adults, potentially leading to repeated activation of the immune system (Wong et al., 2021; Xing et al., 2020; UKHSA, 2022). The SARS-CoV2 envelope glycoprotein S (Spike) contains a sequence with structural motif similar to a bacterial “superantigen” (enterotoxin B from Staphylococcus aureus) (Cheng et al., 2020), capable of directly binding to T cell receptors and triggering excessive and uncontrolled activation of the immune system (Brown and Bhardwaj, 2021; Noval Rivas et al., 2021; Ramaswamy et al., 2021; Sacco et al., 2022). Analogous to HIV-1 patients, children previously infected with SARS-CoV-2 may experience repetitive immune activation due to the prolonged presence of SARS-CoV-2 in the gastrointestinal tract (Brodin, 2022; Xia et al., 2022). Consequently, if, normally, the immune system activates less than 0.001% of the available T lymphocytes, in the presence of a superantigen up to 30% T lymphocytes are activated, generating a massive release of proinflammatory cytokines (Brodin, 2022), similarly to what has been reported for multiple bacterial toxins or viral molecules (Pérez-Gracia et al., 2022). Under these conditions, children may become susceptible to other viral infections, and repeated activation by adenovirus coinfection could increase the risk of toxic shock and liver damage (Yarovinsky et al., 2005; Brodin and Arditi, 2022). This scenario may arise from the interaction between the SARS-CoV-2 superantigen and a host sensitized with HAdV-41F (Brodin and Arditi, 2022). MIS is observed in a small proportion of children, appearing a few weeks or months after disease onset, even in cases of mild disease, leading to hepatitis that requires hospitalization in 40% of cases. It has been suggested that the deterioration of the intestinal barrier associated with these infections allows the viruses to enter the bloodstream, triggering inflammation (Cantor et al., 2020; Yonker et al., 2021). Animal studies have demonstrated that HAdVs infection sensitizes subjects to subsequent staphylococcal enterotoxin B-mediated toxic shock, resulting in liver failure and death. This phenomenon may be attributed to HAdV-induced type 1 immune dysregulation, characterized by excessive production of IFN-γ and IFN-γ-mediated hepatocyte apoptosis (Yarovinsky et al., 2005). Therefore, it is suggested that severe acute hepatitis in children could have a similar mechanism, resulting from HAdVs infection with intestinal trophism in children previously infected with SARS-CoV-2 (Brodin and Arditi, 2022).

Despite initially appearing as an enticing hypothesis, the relationship of the disease with the SARS-CoV-2 infection was not confirmed (Brodin and Arditi, 2022; Pérez-Gracia et al., 2022). Moreover, the recent study by Ho et al., has confirmed that that there is no direct link between COVID-19 and the occurrence of acute hepatitis (Ho et al., 2023), thereby debunking the captivating “superantigen” hypothesis.

## The contribution of HAdV as potential triggers of severe acute hepatitis in children?

Adenovirus infections have been frequently reported in countries where cases of severe acute hepatitis have been documented, with approximately 90% of affected children testing positive for human adenovirus (HAdVs) in the two cohorts from Alabama (USA) and the United Kingdom (UK). Adenoviruses (family *Adenoviridae)* are 90–100 nm non enveloped viruses, with an icosahedral nucleocapsid containing a linear genome (double-stranded, ds) dsDNA that ranges between 26 and 48 Kb. These agents have a broad variety of vertebrate hosts. In humans, more than 50 distinct adenovirus serotypes are recognized associated to illnesses that range from the common cold to life-threatening multi-organ disease in people with a weakened immune system (Lynch et al., 2011). Currently, 88 human adenoviruses (HAdVs) of seven species have been defined (Human Adenovirus A to G) associated with different conditions (Benkő et al., 2022).

Adenovirus serotypes 40-41 have a higher affinity for the gastrointestinal tract among the different adenovirus species, being HAdV-41 infection one of the most frequent causes of viral gastroenteritis in children. These serotypes have been reported in immunosuppressed individuals related to numerous conditions such as hematopoietic stem cell transplantation, solid organ transplantation, human immunodeficiency virus infection, chemotherapy, and congenital hepatitis, or immunodeficiency syndromes (Rocholl et al., 2004; Ozbay Hoşnut et al., 2008; Khalifa et al., 2022). Despite, they are not associated to hepatitis in immunocompetent children (Rocholl et al., 2004; Hough et al., 2005; Kawashima et al., 2015), multi organ infections with HAdVs, including hepatitis, from very focal to very extensive, have been reported occasionally in immunocompetent newborns (Schaberg et al., 2017). In the case of immunocompromised adults, hepatitis caused by these adenovirus is lethal, while in pediatric patients lethality is approximately 60-65% (Ronan et al., 2014).

It is noticeable that all 9 children in the Alabama series and 27 of the 30 children who underwent molecular testing in the UK study, tested positive for HAdV human adenovirus type F41 (HAdV-F41) (Karpen, 2022; UKHSA, 2022). Indeed, HAdV has been detected in whole blood specimens, with the high positivity rate of 69% in EU, suggesting its role as a pathogenic microorganism. According to UK respiratory infection surveillance data, a significant increase in the rate of HAdVs infection in healthy children has been observed in recent weeks compared to previous years, especially in children from 1 to 9 years of age (UKHSA, 2022). Remarkably, serum viral load values of HAdV-F41 in patients with progression to liver failure, especially those who required liver transplantation, were substantially higher compared to patients who recovered spontaneously: median of 20,722 versus 2,733 viral copies/mL (Kelgeri et al., 2022). Altogether these reports represent adequate evidence that HAdV-F41 may have been involved as a cause for pediatric liver failure (Garnett et al., 2009).

However, some questions arise regarding the aforementioned observations. The ECDC report indicates that more than 25% of children with severe acute hepatitis tested positive for HAdVs infection in their respiratory, serum or stool samples (who/ecdc, 2022). However, concerns about the accuracy of these findings are raised due to the detection of positive throat swabs in 11% of healthy children in the same ECDC report. Furthermore, studies have shown that some children may test positive for adenovirus only in whole blood and at very low concentrations (Leen and Rooney, 2005; Djeneba et al., 2007; Song et al., 2016). Histological studies of liver biopsies from affected patients also fail to provide evidence of hepatocellular adenoviral infection (Gutierrez Sanchez et al., 2022). It is important to note that the standard reference method for diagnosing HAdVs-related hepatitis is the detection of the virus in inclusion bodies in liver biopsies (Schaberg et al., 2017), which have not been found in the reported cases mentioned above. Finally, the absence of adenovirus in hepatocytes, but the presence of severe liver injury leading to acute liver failure, may be related to an abnormal immune response of the host’s hepatic immune system(Pérez-Gracia et al., 2022) (Rocholl et al., 2004; Ozbay Hoşnut et al., 2008; Khalifa et al., 2022).

In summary, the previous reports indicate that 90% of affected children tested positive for human adenovirus in two cohorts from USA and the UK. Additionally, adenovirus positivity was found in 55% of cases in Europe and 45% in the USA (World Health Organization (WHO), 2022). This finding provides sufficient evidences that HAdVs may be involved, in some way, in causing severe acute hepatitis in children.

## The contribution of HAdV in association with AAV2 as a cause of severe acute hepatitis in children

AAV2 is a single-stranded DNA virus with 4675 nucleotides which belongs to the family *Parvoviridae, genus Dependoparvovirus* (ICTV, 2023). The AAV2 virus has a broad tissue tropism that can only replicate in the presence of a “helper” virus, often a Human Adenoviruses (HAdV), but also herpesvirus or even human papillomavirus for productive replication in mammalian cells (Casto et al., 1967; Buller et al., 1981; Carter, 2004; Samulski and Muzyczka, 2014). AAV itself did not cause any disease in the absence of helper virus, AAVs can give rise to latent infections where the viral DNA is maintained as circular episomes or is integrated in the chromosomal DNA (Muzyczka and Berns, 2001; Kerr et al., 2006; Samulski and Muzyczka, 2014). Although contact with AAVs is nearly universal, with more than 70% of the population having antibodies to AAV1–3 and AAV5, the presence of viral DNA has been detected in only a small proportion of cases. In fact, AAVs infection has not been linked to any specific human disease and typically elicits a mild immune response. Even immunocompromised patients exhibit very low levels of viremia, and there is limited evidence to suggest that AAVs cause clinical symptoms. These observations indicate that AAVs do not play, by itself, a pathogenic role in organ-specific diseases or in highly immunocompromised populations (Heugel et al., 2011).

Surprisingly, a recent study carried out in Scotland, detected the AAV2 virus in plasma of 9 out of 9 children and in the liver of 4 out of 4 patients, but no positivity was found in serum/plasma of 13 age-matched healthy controls (Ho et al., 2023). In addition, 12 children infected with adenovirus (HAdV) with normal liver function were found negative for AAV, and similarly, the HAdV was not detected in 33 hospitalized children with hepatitis of other etiologies. In this study HAdV (species C and F) and human herpesvirus 6B (HHV6B) have been detected in 6 out of 9 and 3 out of 9 affected cases respectively, including in 3 out of 4 and 2 out of 4 liver biopsies.

In the same report, it was observed that 8 out of 9 patients had the HLA-DRB1* 04: 41 class II allele, present at a much lower frequency in the general Scottish population, indicative of an association of this allele with the increased susceptibility to infection with the above mentioned viruses (Ho et al., 2023).

In this context, prolonged lockdowns have been proposed as a factor limiting children’s exposure to HAdVs and AAV, potentially reducing their natural immunity. Sequencing data indicate no amino acid differences in the E1A, E2A and E4 HAdVs proteins, and no relevant differences have been reported in the AAV2 (AAVv66) capsid that could impact their tropism. Nevertheless, patients displayed some dissimilarities compared to healthy controls (Grand, 2022; Gutierrez Sanchez et al., 2022; Morfopoulou et al., 2022; Ho et al., 2023). On the other hand, alterations in AAVv66 have been observed diminishing its ability to bind heparin. These changes may account for increased virion stability, production, evasion of neutralizing antibodies, enhanced tissue spread, and improved transduction potential to the central nervous system (Hsu et al., 2020; Ho et al., 2023).

The evidence for the presence of HAdV41 in many cases of severe acute hepatitis in children is strong, and recent studies provide unequivocal confirmation of AAV2 involvement. These findings suggest that HAdVs may potentially act as a helper virus for AAV2 (Leiskau et al., 2023), with/without eventual mutations in the AAV2 genome (Grand, 2022; Morfopoulou et al., 2022; Ho et al., 2023).

Technological solutions based on high-throughput sequencing methodologies have improved the chances of detecting DNA and RNA viruses in a clinical sample. Using metagenomic (Ibañez-Lligoña et al., 2023) to identify both RNA and DNA viruses next-generation sequencing (NGS) on NextSeq500 (Illumina) platform, and target enrichment NGS using VirCapSeq-VERT Capture probes, together with reverse transcription-polymerase chain reaction (RT-PCR), serology and *in situ* hybridisation(ISH), Ho et al. detected recent infection with AAV2 in the plasma and liver samples of 81% of the Scottish pediatric hepatitis cases and only in 7% of controls. Interestingly, an increase in HAdV diagnoses in Scotland directly preceded the outbreak of unknown severe hepatitis in children of a similar age (UKHSA, 2022). A helper virus is required to support AAV2 replication, and AAV2 RNA was detected within ballooned hepatocytes suggesting the presence of replicating virus. Moreover, authors found a strong association between affected children and the Human Leucocyte Antigen (HLA) class II DRB1*04:01 allele, since it was identified in 93% (25/27) cases compared with the background frequency of 10/64 (16%) in study controls (Ho et al., 2023), and only 0.11% in UK unrelated biobank samples.

## To summarize

Regarding the etiology of Pediatric Severe Acute Hepatitis, recent publication emphasizes that there is no link between COVID-19 and the current outbreak, contradicting previous suggestions. Autoimmune disease is also deemed less likely due to the absence of autoantibodies in affected cases and atypical histology in liver specimens. Results from Ho et al. strongly indicate a plausible association between concurrent HAdV infection and coinfected or reactivated AAV2 infection, leading to severe acute hepatitis in susceptible children carrying the HLA class II allele HLA-DRB1*04:01. This study paves the way for population-level investigations into the role of AAV2 and a helper virus (such as HAdV and/or HHV6B) of pediatric severe acute hepatitis with an unknown etiology.

## Author contributions

The three authors FR-F, JQ and AR-S have significantly contributed in designing, collecting information and writing the manuscript. All authors approved the submitted version.
